# The experience of surgical cancer patients during the COVID-19 pandemic at a large cancer centre in London

**DOI:** 10.1007/s00520-024-08528-w

**Published:** 2024-05-01

**Authors:** Beth Russell, Hajer Hadi, Charlotte L. Moss, Saran Green, Anna Haire, Harriet Wylie, Jasmine Handford, Maria Monroy-Iglesias, Harvey Dickinson, Kate Haire, Mieke Van Hemelrijck

**Affiliations:** 1 Translational Oncology and Urology Research, School of Cancer and Pharmaceutical Science, King’s College London, London, UK; 2 South-East London Cancer Alliance, London, UK

**Keywords:** COVID-19, Coronavirus, SARS-CoV-2, Patient experience, Cancer, Surgery, Cancer surgery

## Abstract

**Background:**

The COVID-19 pandemic has had an enormous impact on the experiences of patients across all health disciplines, especially those of cancer patients. The study aimed to understand the experiences of cancer patients who underwent surgery during the first two waves of the pandemic at Guy’s Cancer Centre, which is a large tertiary cancer centre in London.

**Methods:**

A mixed-methods approach was adopted for this study. Firstly, a survey was co-designed by the research team and a patient study group. Patients who underwent surgery during the COVID-19 pandemic were invited to take part in this survey. Results were analysed descriptively. Three discussion groups were then conducted to focus on the main themes from the survey findings: communication, COVID-19 risk management and overall experience. These discussion groups were transcribed verbatim and underwent a thematic analysis using the NVivo software package.

**Results:**

Out of 1657 patients invited, a total of 250 (15%) participants took part in the survey with a mean age of 66 (SD 12.8) and 52% females. The sample was representative of a wide range of tumour sites and was reflective of those invited to take part. Overall, the experience of the cancer patients was positive. They felt that the safety protocols implemented at the hospital were effective. Communication was considered key, and patients were receptive to a change in the mode of communication from in-person to virtual.

**Conclusions:**

Despite the immense challenges faced by our Cancer Centre, patients undergoing surgery during the first two waves of the COVID-19 pandemic had a generally positive experience with minimal disruptions to their planned surgery and ongoing care. Together with the COVID-19 safety precautions, effective communication between the clinical teams and the patients helped the overall patient experience during their surgical treatment.

**Supplementary Information:**

The online version contains supplementary material available at 10.1007/s00520-024-08528-w.

## Introduction

The COVID-19 pandemic has had profound consequences for the health of people globally. It continues to present a challenge to public and global health and has had a significant impact on the delivery of care for cancer patients globally [1, 2]. The UK has been severely impacted by the pandemic, resulting in one of the highest confirmed death rates in the world in 2020 [3]. A survey of members of the public found that access to health services for people with long-term health conditions such as cancer and mental health was 20% lower during the pandemic compared to pre-pandemic times [4].

A national report from the NHS Patient Survey Programme was published in November 2020 regarding the inpatient experience during the early stages of the pandemic (April–May 2020) [5]. The results suggested that the experiences of inpatients during this time were generally positive, with 83% of respondents saying they felt safe from the risk of catching COVID-19 in the hospital. In the context of cancer specifically, however, few studies examined the experience of patients.

A study by Monroy-Iglesias et al. [6] reporting on the outcomes of cancer patients receiving radical surgery with curative intent during the first wave of the COVID-19 pandemic stated that the implemented COVID-19 minimal pathways (where patients self-isolated prior to admission and were swabbed for COVID-19 within 72 h of surgery) were safe for cancer patients requiring surgical treatment, with very few COVID-19 infections. The authors suggest that it is critical for all elective cancer surgeries to return to their normal levels of functioning to avoid future complications due to delays in oncological care.

The aim of the current study was to look specifically at the experiences of cancer patients who had undergone surgery during the first two waves of the pandemic at a large cancer centre in the UK.

## Methods

### Study participants

Patients were invited to take part in the study if they had a diagnosis of cancer and underwent surgery (for any reason) for their cancer during wave one (April–September 2020) or wave two (January–April 2021) of the COVID-19 pandemic.

### Survey design and distribution

A survey was co-designed by the research team and a patient study group. The patient study group was also involved in the development and validation of the patient information sheet. Patients were invited to participate in the survey through a posted letter. This letter outlined the research aims and included a consent form where patients could either choose to complete the survey online, via phone or via paper. The online survey was hosted using REDCap which is a secure online application for building and managing online surveys and databases.

Service evaluation approval for the survey was granted through Guy’s and St Thomas NHS Foundation Trust. The survey was open from 1st February to 29th April 2022.

### Focus groups

Potential patient participants were identified using snowball sampling from which a total of 16 patients agreed to participate. Three discussion groups were carried out, two of which were undertaken online, using Microsoft Teams, and one was in person. Focus group discussions were guided by the results of the survey and were based on identified emerging themes (communication, COVID-19 risk management and overall experience).

### Analysis

The results of the survey were analysed using descriptive statistics. The three discussion groups were transcribed verbatim. The transcriptions were then analysed in a deductive manner based on the three themes identified from the analysis of the survey results, as mentioned above. During this deductive analysis, we looked for quotes in support of these three key themes.

## Results

### Survey respondent cohort characteristics

A total of 1657 patients were invited to take part in the survey. Responses were received from 250 (15%) patients. Most survey participants (71%) were aged 60 and over, which is reflective of the cohort of patients invited (Table 1). There were slightly more female survey participants (52%) than males (47%). A large proportion (90%) of the survey participants were of White ethnicity, and the second largest proportion of patients (5%) was of Black ethnicity.

**Table 1:** Survey participant demographics

	*N* (total *n* = 250)	%
Age groups		
20–29	1	0.40
30–39	12	4.80
40–49	17	6.80
50–59	40	16.00
60–69	71	28.40
70–79	81	32.40
≥80	25	10.00
Missing	3	1.20
Sex		
Male	117	46.80
Female	131	52.40
Missing	3	1.20
Ethnicity		
White	225	90.00
Mixed	3	1.20
Asian	6	2.40
Black	12	4.80
Other	0	0.00
Missing	4	1.60
Cancer		
Brain	2	0.80
Head and neck	32	12.80
Lung	67	26.80
Upper gastrointestinal	12	4.80
Lower gastrointestinal	14	5.60
Prostate	27	10.80
Bladder	19	7.60
Renal	22	8.80
Testicular	2	0.80
Breast	37	14.80
Pancreatic	1	0.40
Neuroendocrine	1	0.40
Gynaecological	19	7.60
Skin	20	8.00

In terms of cancer types, the largest represented group of the study participants were urology (28%) and thoracic/lung cancer patients (26%). This is reflective of those invited, in which urology made up 23% of the population whilst thoracic/lung patients made up 26% of the population invited. The next largest groups of study participants were breast cancer (15%) and head and neck cancer patients (14%). Forty percent of patients with skin cancer (*n* = 20) invited to take part in the survey did so, whilst 100% (*n* = 1) of neuroendocrine patients participated.

### Communication

**Fig. 1:**
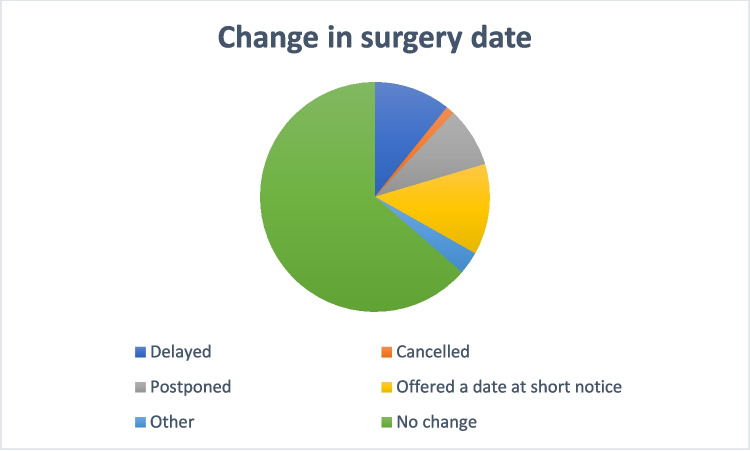
Change in surgery date

One hundred survey participants (40%) reported having been made aware of changes to their planned date for their cancer surgery. Nineteen percent stated that their surgery had been delayed or postponed to a later date. Thirteen percent said they were offered another date at short notice, whilst 1% had their surgery cancelled altogether (Fig. 1). A large proportion of survey participants (83%) expressed that they did not want the date of their surgery delayed through concerns of catching COVID-19. The majority of survey participants either agreed (29%) or strongly agreed (58%) that they were happy with the explanations given to them about their surgery plan.

Most participants (95%) said they felt supported by the staff at the cancer centre in dealing with their cancer. In terms of contact regarding the planning of their surgery, participants reported being contacted through several methods, with telephone calls (71%) seeing the highest proportion of responses. This was followed by letters (57%) and in-person visits (50%). Nine percent of respondents reported using a video appointment during the planning of their surgery. The survey showed that even though a large proportion reported no change in attitude towards video appointments (45%), 17% said they were more receptive to video appointments, and 7% felt more secure at these types of appointments. Most respondents reported that in the future they would either prefer to have in-person appointments (48%) or a mixture of in-person and video appointments (43%).

When asked about aftercare, 94% of respondents said they had received some sort of practical advice and support in dealing with the immediate side effects of their surgery, with another 1% claiming they did not need any. Most respondents reported that the information they were given was easy to understand (88%); however, a small proportion reported that their information was difficult to understand (8%). Eleven percent of respondents reported that they did not know who to contact after their discharge. Overall, a large proportion of patients felt the information provided to them upon discharge from the hospital was either good (38%) or excellent (52%).

### COVID-19 risk management

Almost all study participants had received at least one COVID-19 vaccination by the date of surgery (96%), with the majority of those having received three doses (92%). The study participants were made aware of a variety of COVID-19 safety measures, with most reporting an assessment of health status via telephone appointment prior to admission (66%), pre-operative isolation period (72%), negative COVID-19 test before surgery (85%), admission the night before surgery (60%) and no visits allowed from friends or family (74%). Less than a third (32%) of respondents reported feeling afraid of catching COVID-19 whilst in hospital.

### Overall experience

When asked whether the whole team looking after the patient worked well together to give them the best possible care, almost all respondents said ‘yes’ (92%). Just under 5% of respondents answered ‘no’ to this question. In terms of the administration of their care, 85% of respondents rated this as good, 10% as fair and 4% as poor. Finally, when asked to rate their overall care on a scale of one to ten, over 90% of respondents answered with an eight or above.

### Patient discussion groups

Sixteen patients were involved in the patient focus groups comprising three head and neck, one head and neck and lung cancer, four prostate, four bladder and four breast cancer patients. In total, there were nine male and seven female patients. The themes identified from the survey results were used to guide the deductive analysis. The themes identified were communication, COVID-19 risk management and overall experience.

Under the communication theme, the patients discussed the issues around communication with family during their time in the hospital as well as the communication with the hospital and clinical staff before, during and after their surgery, and how COVID-19 impacted this communication. With regards to communication with family, there were consistent reports of concerns and difficulties with access to phones and/or use of personal mobiles. Several patients also reported slow communication with family members post-surgery to let their loved ones know they were ok.

“I had my phone but my partner was waiting at home. They said that they were going to call him when I was out of surgery, and they didn’t call and he couldn’t get reach of anybody. He was calling at all times of the day, just to try and talk to somebody on the ward and he never got in touch with someone which was a bit of a shame, because he was a bit worried”. [Participant 12, breast cancer]

“When you’re coming around in the recovery room it can be a bit slow. I think it would be nicer if they could have communicated to the family”. [Participant 15, head and neck cancer]

Communication with the hospital in the lead-up to the surgery was generally very positive with patients saying they favoured the mix of email and telephone calls with some face-to-face appointments even during the peak of the COVID-19 pandemic.

“I did like having the option of a call sometimes, because I like to be able to ask questions and talk through things, but in terms of information and needing to know things, e-mail for sure so that I like to read and reread and then research and then it calms my anxiety.” [Participant 12, breast cancer]

Patients felt well-supported by their clinical teams. This was mainly down to the level of communication between themselves and the hospital staff.

“I could phone up the two main nurses anytime… it’s so easy for me to go in. I can just walk in and talk to anyone, and walk out which they don’t mind and which is good”. [Participant 15, bladder cancer]

“I have had all the communications - everything; so face to face, e-mail, phone calls, video-calls. So yeah, I had a bit of everything. I can say that yeah, I was really impressed” [Participant 10, breast cancer]

Patients reported being happy with the COVID-19 precautions taken by the hospital staff, with several patients saying it made the pre-operative process more efficient.

“…all my pre-op’s and everything was done at that same appointment, I didn’t have to keep coming back and forth. So that was a plus.” [Participant 11, head and neck and lung cancer]

“I have to agree with that comparing experience during post-pandemic and before things are far more efficient, and happened quicker. I love phone consultations when it’s not needed. So, overall I think my experience has been much better post pandemic than before.” [Participant 9, prostate cancer]

“…when I had to go in and all the precautions and stuff and there was very minimal delay and I was, you know, quite surprised how well it was all being handled in the Cancer Centre at Guys so. I felt lucky to be going to that one to be honest.” [Participant 10, breast cancer]

The overall experience of patients was generally very positive with adjectives such as ‘outstanding’ and ‘remarkable’ being used by the patients to describe their experiences.

“I didn’t feel like it was a pandemic at all, so everything went smoothly. No issues at all.” [Participant 13, bladder cancer]

“It was really outstanding.” [Participant 12, breast cancer]

“It was wonderful for me. Considering the sessions that that they were. They were remarkable.” [Participant 2, bladder cancer]

## Discussion

This study, utilising both a survey and patient discussion groups, found that overall, the patient experience of surgical patients during the COVID-19 pandemic was a very positive one. Conversations within the discussion groups supported the data gathered from the survey reflecting that patients were happy with the COVID-19 safety precautions adopted by the hospital, and they appreciated the flexibility of having both virtual (whether this be email, phone call or video calls) and face-to-face appointments.

COVID-19 put an immense strain on already stretched surgical waiting lists. At our centre, a COVID-safe pathway was implemented (described elsewhere [6, 7]) and aimed to continue the care of cancer patients whilst mitigating the risk of COVID-19 infection. In our survey, less than a third of patients were afraid of catching COVID-19 whilst in hospital for their surgery. These results echo those of another study carried out in urological patients in the US, who also felt that when robust and effective precautions and protocols were put in place, the fear of COVID-19 infection was reduced [8]. Moreover, a study in China which surveyed patients undergoing irritable bowel disorder (IBD)-related surgery found that despite a proportion of patients feeling some level of anxiety about the impact of COVID-19 on their surgical outcomes, with the right precautions in place, the overall experience was still very good for the majority of surgical patients [9]. Our previous studies have shown that the provisions implemented at our Cancer Centre ensured that patients continued to be treated in a safe and effective manner [6, 7]. This current study further delved into the experience of these patients, and the results show that less than one in five patients reported a change in their original surgery date due to the pandemic, and only 1% of patients had their surgery cancelled. Moreover, patients still felt supported throughout their surgical experience and felt they were still involved in the decision-making process around their treatment.

Effective communication is an essential aspect of clinical care in cancer patients. Results from our survey and discussion groups suggest that our centre had effective communication with patients throughout all aspects of the patient journey, including the transition from in-person to virtual consultations. Numerous studies have demonstrated the significance of communication and how this is linked to patient experience [10, 11]. Specifically, the study by Odai-Afotey et al. [10] adopted a mixed-methods approach to look at patient experience in oncology at their centre. Their analysis identified two main themes—hospital experience and physician communication skills, much the same as the current study. Physician attentiveness or lack thereof was identified as a defining aspect of shared decision-making and thus patient experience. In the future, it may be beneficial to repeat a similar study to the current study to compare the quality of communication during and after the pandemic. A tool which may be of use in such a study is the EORTC QLQ-COMU26 communication questionnaire [17].

Despite the overarching positive physician communication reported by the patients at our Centre, as discussed within the focus groups, one possible area for improvement is the communication with family members in the post-operative setting whilst patients are unable to contact their loved ones themselves. It should however be appreciated that this may have been particularly troublesome during the pandemic when clinical teams were stretched and were not prioritising communication with the patients’ family members.

Around a quarter of patients in our survey reported either feeling more secure at video appointments and/or feeling more receptive to video appointments compared to pre-pandemic. Whilst some patients would still prefer to have all face-to-face appointments, a large proportion also gave a preference over having a mixture of face-to-face and virtual consultations. These results were echoed by another study on lung cancer patients in New Zealand [12]. Results from that study found that overall, telehealth was well-received by patients, but there was still a small proportion of patients who placed a huge value on the continuity of face-to-face appointments. This shows that whilst some patients are reluctant to change, there is also a large proportion of patients who are willing to make changes and are receptive to changes in the mode of contact with their clinical teams. A couple of recent studies conducted on cancer patients during the COVID-19 pandemic also described high levels of acceptability of telemedicine [13, 14]. Whilst in the study by Boehm et al. [14], all telemedicine referred to videoconferences, the study by Burbury et al. [13] investigated more than one method of telemedicine and reported a preference for video compared to phone appointments.

This study benefitted from a mixed methods approach utilising both a survey and a series of discussion groups to delve into the factors affecting the surgical patient experience over COVID-19. The use of the discussion groups allowed the themes and results from the survey to be investigated in further detail. Furthermore, there was a good representation of patients from across a variety of tumour types and it is reflective of the tumour types in the overall patient cohort at our Cancer Centre. The overall response rate for the survey was 15% which was lower than was aimed for; however, it is worth noting that patients were subjected to many surveys during COVID times as this was the preferred research method and hence may have experienced survey fatigue. Furthermore, our survey may have been subject to nonresponse bias as patients who take part in the survey may be more likely to report negative experiences, a phenomenon often observed in surveys [15, 16].

Despite our Cancer Centre sitting in an ethnically diverse area of London, the overwhelming majority of the patients surveyed were of White ethnicity with other ethnicities making up just 8% of the total population. This over-representation of White ethnicity regularly occurs within clinical research and poses a significant problem [17]. The lack of diversity in our ethnicity data meant we were unable to look at possible inequalities surrounding ethnicity and patient experience. Other studies which have looked into this, such as that by Llanos et al. [18] in the US, concluded that race and ethnicity were associated with delayed or discontinued treatment and longer delays to restart cancer treatment after COVID-19.

Despite the immense pressure and challenges faced by the clinical teams at our Cancer Centre over COVID-19, results from this study suggest that with the robust protocols and effective communication, the overall patient experience was still generally very positive with minimal disruption to the care of surgical patients.

### Supplementary Information

Below is the link to the electronic supplementary material.

Supplementary file1 (PDF 71 KB)

## Data Availability

The data presented in this study are available upon request from the corresponding author. The data are not publicly available due to ethical reasons.
