# Supplementary material for: The experience of surgical cancer patients during the COVID-19 pandemic at a large cancer centre in London
Source: Support Care Cancer. 2024 May 1;32(5):321. doi: 10.1007/s00520-024-08528-w (PMC11062958; doi:10.1007/s00520-024-08528-w)
Supplement: Supplementary file 1 — Supplementary file1 (PDF 71 KB) [file 520_2024_8528_MOESM1_ESM.pdf]

Cancer patient experience during the COVID-19 pandemic: Service Evaluation Study on Cancer Patient's Postoperative Experience during the COVID-19 Pandemic

Dear Participant,

Thank you for showing interest in taking part in our service evaluation project.

Before you decide to take part, we would like you to understand why the service evaulation is being done and what it will involve. Please read the following information carefully.

Your participation in this questionnaire will not in any way affect your treatment and nothing you say will be disclosed to the healthcare staff looking after you.

You have the right to stop the questionnaire or withdraw from the project at any time. The information obtained here will be strictly confidential; that means that nobody will be able to identify you or other personal information.

Investigating the experiences of Cancer Patients undergoing Surgery during COVID-19 pandemicThe COVID-19 pandemic has had profound consequences for the health of people globally. It has led to a dramatic loss of human life worldwide and is the defining global health crisis in our current times. COVID-19 still presents a challenge to public and global health causing millions of deaths and is having a significant impact on the delivery of care and impacting cancer patients globally.

We are investigating cancer patients’ experiences during the first and second wave of the pandemic, whilst looking deeper into whether patient experiences were impacted by health inequalities during this period.

Your participation in this service evaluation project will help to ensure that cancer patients receiving safe, high-quality care during these difficult times.

What is the purpose of the study?

This questionnaire aims to understand the experience of people who had cancer surgery during the COVID-19 pandemic. The questionnaire asks about your experience of stressful events, concerns about risk, wellbeing, and coping. Your views may help the NHS to monitor and improve the quality of cancer services so that they better meet the needs of patients.

More specifically, we would like to learn more about:

Your experience of cancer care during the pandemicCancer patients’ perceptions about COVID-19Whether access to cancer care was impacted for cancer patients during the pandemicHow patients were impacted by health inequalities; unfair and avoidable differences in health that arise because of the conditions in which we are born, grow, live, work, and ageWhy have I been invited?

You have been selected because you have had a surgery to treat cancer in South East London during the COVID-19 pandemic.

Do I have to take part?

No, taking part is voluntary. You are free to decide whether you would like to join the study or not.

If you agree to take part, we will ask you to read the consent form below and then proceed to the questionnaire.

If you do not wish to take part, you will not be asked to do anything else. This will not affect the quality of care you receive from Guy’s Cancer Centre.

Thank you for taking the time to read this information.

Should you have any further questions, please contact Miss Hajer Hadi on: Hajer.hadi@gstt.nhs.uk or cancerdata@gstt.nhs.uk or by telephone: 07548153078.

Informed consent:
By completing this questionnaire you are agreeing:

This consent form is to ensure that I understand the purpose of my involvement in the study/service evaluation and that I agree to the conditions of my participation, to have read the accompanying information sheet and certify that I approve the following:

I am voluntarily taking part in this project, and I am aware that I am free to withdraw at any point without giving a reason, and without my medical care or legal rights being affected. I understand that if I do withdraw, my data may not be erased but will only be used in an anonymised form as part of an aggregated dataset. I know how to contact the research team if I need to. I understand that the personal data collected from me during the course of the project will be used for the purposes outlined above in the public interest. That my personal information can be held and used by Guy's & St Thomas' NHS Foundation Trust (GSTT), South East London Cancer Alliance (SELCA) or NHS England and organisations acting under its instructions. I do not expect to receive any benefit or payment for my participation. I have been able to ask any questions I might have, and I understand that I am free to contact the researcher with any questions or concerns I may have in the future. The information I have provided will be treated in confidence by the researcher and my identity will be protected in the publication of any findings. Any variation of the conditions above will only occur with my further explicit approval. I understand that the confidentiality of the data I donate, and information derived will be protected and pseudonymised. I have been told that all medical information / data pertaining to me will be protected by the principles of confidentiality and both national and EU data protection legislation. I give permission to include my pseudonymised data in future cancer research studies. This may include studies which look at my data but does not impact my care. I give permission to be contacted regarding participating in an interview or focus group as part of the cancer patient experience during the COVID-19 pandemic. I understand my participation is voluntary, and any contribution I make within these sessions will be anonymised.

Taking part in this survey is voluntary.

---

1) If you agree to this, please tick this box ☐ Yes

Cancer Patient Experience during the COVID-19 pandemic

This survey takes approximately 10-20 minutes.

This survey asks about your views on preparation, experience of stressful events, concerns about risk, wellbeing, and coping. Your views will help the NHS monitor and improve the quality of cancer services so that they better meet patient needs. Your participation in this project will help us to ensure that cancer patients are receiving continued adequate and effective care.

Tell us your experience.

This questionnaire is divided by eight sections:About youYour perceptions of COVID-19 before your surgeryYour care before surgeryYour surgery & hospital stayContact & communicaton with you during your treatmentYou care after discharge and follow-upYour overall care experienceMore about you – understanding health inequalities

2) Today's date ______________________________

3) Forename: ______________________________

4) Surname: ______________________________

5) Date of birth ______________________________

6) Sex
- ◯ Male
- ◯ Female
- ◯ Other
- ◯ Prefer not to say

7) Postcode: ______________________________

Section 1: About you

Question	Response
Type of cancer/cancer diagnosis:	☐ Brain<br>☐ Head & Neck (e.g. mouth, thyroid, throat)<br>☐ Lung<br>☐ Haematological (blood cancer, leukaemia)<br>☐ Upper Gastrointestinal (e.g., oesophagus, stomach, liver)<br>☐ Lower Gastrointestinal (e.g. bowel, rectum)<br>☐ Prostate<br>☐ Bladder<br>☐ Renal (kidney)<br>☐ Testicular<br>☐ Breast<br>☐ Pancreatic (tissues of the pancreas)<br>☐ Neuroendocrine (nerve and hormone-producing cells)<br>☐ Gynaecological (e.g., uterus, cervical, ovarian)<br>☐ Sarcoma (bone or muscle)<br>☐ Skin<br>☐ Other
If other, please specify	__________________________________
What cancer treatments did you receive between 'April 2020 and April 2021'? (Select all that apply)	☐ Chemotherapy<br>☐ Radio/radiation therapy (e.g., brachytherapy, external beam radiotherapy)<br>☐ Surgery<br>☐ Immunotherapy<br>☐ Hormonal therapy<br>☐ Targeted therapy (oral drugs)<br>☐ Other
If other, please specify	__________________________________
Have you had a COVID-19 vaccination?	◯ Yes<br>◯ No<br>◯ Prefer not to say
If yes, how many doses?	◯ 1<br>◯ 2<br>◯ 3

Section 2: Your perceptions of COVID-19 before your surgery

These questions are about COVID-19 and how it affected you when preparing for your cancer surgery.

These questions are related to your thoughts, knowledge, and perspectives on the pandemic.

---

14) In your opinion, do you believe your risk of contracting COVID-19 is:
- ◯ Lower than the general population
- ◯ Higher than the general population
- ◯ The same as the general population

---

15) I am more afraid of COVID-19 than my cancer.
- ◯ Strongly agree
- ◯ Agree
- ◯ Neither agree nor disagree
- ◯ Disagree
- ◯ Strongly disagree

---

16) I was afraid of getting COVID-19 infection while visiting hospital or during my hospital stay.
- ◯ Strongly agree
- ◯ Agree
- ◯ Neither agree nor disagree
- ◯ Disagree
- ◯ Strongly disagree

Section 3: Your care before surgery

These questions are about how you were referred before your surgery.

Were you referred by your GP?	◯ Yes, I was referred by my GP practice<br>◯ No, I wasn't referred by my GP practice<br>◯ Not applicable
If no, for what reason?	◯ GP surgery was closed<br>◯ GP practice had no access to video conferencing/telephone appointment<br>◯ No timely appointments available<br>◯ I did not want to visit my GP during the pandemic<br>◯ I did not need to<br>◯ None of the above

These questions are about how you were supported before your surgery

These are related to your worries, needs and support from the hospital staff. These might have been your physical health, practical, psychological, or social needs.

How comfortable are you seeking healthcare services from the following?

	Extremely comfortable	Very comfortable	Somewhat comfortable	Not comfortable at all
Your GP practice	◯	◯	◯	◯
Your local hospital	◯	◯	◯	◯
Accident & Emergency or Urgent Care facility	◯	◯	◯	◯

	Strongly agree	Agree	Neither agree nor disagree	Disagree	Strongly disagree
I was concerned about not being able to visit my surgeon or cancer doctor during COVID-19 lockdown?	◯	◯	◯	◯	◯

Did you have a main contact person in the team looking after you, such as a clinical nurse specialist, who would support you through your treatment?	◯ Yes, it was a clinical nurse specialist / cancer nurse<br>◯ Yes, it was another member of the team<br>◯ No<br>◯ Don't know / I can't remember
Were you involved as much as you wanted to be in decisions about your treatment options?	◯ Yes, definitely<br>◯ Yes, to some extent<br>◯ No<br>◯ Don't know / can't remember
Do you feel you received the right amount of guidance and support with your overall health and well-being from the hospital staff?	◯ Yes, definitely<br>◯ Yes, to some extent<br>◯ No<br>◯ Don't know / not applicable

Section 4: Your surgery and hospital stay

These questions are related to your overall experience with the plan for your cancer surgery.

Were you made aware of any changes to the planned date of your cancer surgery due to the COVID-19 pandemic?
- ◯ Yes
- ◯ No
- ◯ I don't know / I can't remember

If yes, how was your surgery date changed?
- ◯ It was delayed
- ◯ It was cancelled
- ◯ It was postponed
- ◯ You were offered a date at short notice
- ◯ Other

How did it make you feel?

____

	Strongly agree	Agree	Neither agree nor disagree	Disagree	Strongly disagree
I wanted to delay the date of my surgery because I was worried about catching COVID-19	◯	◯	◯	◯	◯

	Strongly agree	Agree	Neither agree nor disagree	Disagree	Strongly disagree
I was happy with the explanations given to me about my surgery plan	◯	◯	◯	◯	◯

Which of these COVID-19 safety measures were you aware of? Select all that apply
- ☐ Assessment of health status via telephone triage prior to admission
- ☐ Different hospital location for the surgery
- ☐ Different clinician or surgeon
- ☐ Strict pre-operative self-isolation period up to 14 days with no prior contact with anyone
- ☐ Negative COVID-19 test before surgery
- ☐ Admission the night before surgery
- ☐ Family and friends (no visitors allowed)

How did you feel about them?

____

Could you get help from staff on the ward when you needed it?
- ◯ Yes, always
- ◯ Yes, sometimes
- ◯ No
- ◯ Don't know / can't remember

Do you have any other comments about your surgery or hospital stay?

____

During your hospital stay, could you talk with hospital staff about your worries and fears if you needed to?
- ◯ Yes, always
- ◯ Yes, sometimes
- ◯ No
- ◯ Don't know / can't remember

In general, how would you rate the care you received during your hospital stay?

- ◯ Excellent
- ◯ Good
- ◯ Fair
- ◯ Poor

Section 5: Contact & communication with you during your treatment

This section relates to the main method of contact and delivery of information with your surgery team.

Did you understand the plan of your cancer surgery or what it entailed?	◯ Yes, definitely<br>◯ Yes, to some extent<br>◯ No<br>◯ Don't know / Not applicable
Did you feel supported by the staff of the cancer centre in dealing with your cancer treatment?	◯ Yes, definitely<br>◯ Yes, to some extent<br>◯ No<br>◯ Don't know / Not applicable

This section relates to the main method of contact and delivery of information with your surgery team.

How were you contacted about the planning of your surgery? Select all that apply	☐ Via e-mail<br>☐ Via text messages<br>☐ Via telephone call/conference<br>☐ Via video appointments<br>☐ Via letters<br>☐ In-person visit
If you participated in an online appointment for the planning of your surgery, how was your overall experience of your online appointment?	◯ Excellent<br>◯ Good<br>◯ Fair<br>◯ Poor
Since the start of the COVID-19 pandemic, how has your attitude towards video appointments changed?	◯ I felt more secure at video appointments<br>◯ I am less receptive to video appointments<br>◯ My attitude has not changed towards video appointments<br>◯ I am more receptive to video appointments
In the future, would you prefer phone/video appointment or in-person appointment?	◯ Phone or video appointment<br>◯ In-person appointment<br>◯ Mixture of the two
What is the reason for your preference?	◯ Less time-consuming<br>◯ Convenience<br>◯ Concern for communication of infectious diseases<br>◯ Other
If other, please specify	__________________________

Section 6: Your care after discharge and follow-up

This section relates to your discharge plan and after-care.

Were you offered practical advice and support in dealing with the immediate side effects of your surgery?	◯ Yes, always<br>◯ Yes, to some extent<br>◯ No, but I needed it<br>◯ No, I didn't need it<br>◯ Don't know / can't remember
Did the hospital staff give you information about surgery after-care i.e., what you should or should not do after leaving hospital?	◯ Yes, and it was easy to understand<br>◯ Yes, but it was difficult to understand<br>◯ No<br>◯ Don't know / can't remember
Did you know who to contact after your discharge?	◯ Yes<br>◯ No
If yes, please specify	__________________________________
How would you rate the information provided on your discharge from the hospital?	◯ Excellent<br>◯ Good<br>◯ Fair<br>◯ Poor
After your surgery, how was the first follow-up appointment delivered by your surgical team?	◯ It was done through video appointments e.g., Teams/Zoom/BlueJeans<br>◯ Phone calls<br>◯ It was done in-person with face-to-face appointments

Section 7: Your overall care experience

This section includes care received from your surgeons, doctors, nurses, and other hospital staff.

51) Did the whole team looking after you work well together to give you the best possible care and service?
- ◯ Yes
- ◯ No
- ◯ Don't know / I can't remember

	Good	Fair	Poor	Don't know / can't remember
52) Overall, how would you rate the administration of your care (arranging appointments, getting letters sent to your GP, etc.)?	◯	◯	◯	◯

	1	2	3	4	5	6	7	8	9	10
53) Overall, how would you rate your care on a scale of 1 to 10 with 1 being very poor and 10 being excellent?	◯	◯	◯	◯	◯	◯	◯	◯	◯	◯

54) Was there anything that could have been improved?

____

Section 8: More about you - understanding health inequalities

These questions are related to more details about your overall health.

What is the highest grade or level of school that you have completed?	◯ Secondary school up to 16 years<br>◯ Higher or secondary or further education (A-levels, BTEC, etc.)<br>◯ University/undergraduate degree<br>◯ Post-graduate degree<br>◯ Prefer not to say
What is your employment status?	◯ Self-employed<br>◯ Employed (full time)<br>◯ Employed (part time)<br>◯ Unemployed<br>◯ Retired<br>◯ Other
If other, please specify	__________________________________
Is English your first language?	◯ Yes<br>◯ No
What is your ethnic group?	◯ White<br>◯ Mixed / Multiple ethnic groups<br>◯ Asian or Asian British<br>◯ Black / African / Caribbean / Black British<br>◯ Other ethnic group
If White,	◯ English / Welsh / Scottish / Northern Irish / British<br>◯ Irish<br>◯ Gypsy or Irish Traveller<br>◯ Any other White background
If Mixed / Multiple ethnic groups,	◯ White and Black Caribbean<br>◯ White and Black African<br>◯ White and Asian<br>◯ Any other Mixed / multiple ethnic background
If Asian or Asian British,	◯ Indian<br>◯ Pakistani<br>◯ Bangladeshi<br>◯ Chinese<br>◯ Any other Asian background
If Black / African / Caribbean / Black British,	◯ African<br>◯ Caribbean<br>◯ Any other Black / African / Caribbean background
If Other ethnic group,	◯ Arab<br>◯ Any other ethnic group

Do you have any of the following health conditions? Please select all that apply.

- ☐ Hypertension (high blood pressure)
- ☐ Cardiac (heart disease)
- ☐ Lung disease (respiratory, difficulty breathing)
- ☐ Diabetes Mellitus
- ☐ Mobility problems
- ☐ Other

If other, please specify

____

Smoking status

- ◯ Never smoked
- ◯ Used to smoke
- ◯ Smoke now
- ◯ Prefer not to say

What is your BMI (Body Mass Index)?

- ◯ 18 or less
- ◯ 19-24
- ◯ 25-29
- ◯ 30-34
- ◯ 35+
- ◯ Prefer not to say

These questions are related to your experience in accessing health care services.

	Never	Rarely	Sometimes	Often
How often, if ever, have you personally experienced discrimination or prejudice during your hospital stay?	◯	◯	◯	◯

When receiving care, have you ever had any of the following things happen to you?

- ☐ I have been treated with less courtesy and respect than others
- ☐ I have had received poorer service or care than others
- ☐ People act as if they think you are not smart
- ☐ People act as if they think you are dishonest
- ☐ People act as if they are better than you

What do you think is the main reason for these experiences?

____
